# Structural insights from random mutagenesis of *Campylobacter jejuni* oligosaccharyltransferase PglB

**DOI:** 10.1186/1472-6750-12-67

**Published:** 2012-09-24

**Authors:** Julian Ihssen, Michael Kowarik, Luzia Wiesli, Renate Reiss, Michael Wacker, Linda Thöny-Meyer

**Affiliations:** 1 Empa, Swiss Federal Laboratories for Materials Science and Technology, Laboratory for Biomaterials, CH-9014, St. Gallen, Switzerland; 2 GlycoVaxyn AG, CH-8952, Schlieren, Switzerland

**Keywords:** glycosylation, oligosaccharyltransferase, PglB, *Campylobacter jejuni*, random mutagenesis, screening, ELISA, directed evolution, conjugate vaccine

## Abstract

**Background:**

Protein glycosylation is of fundamental importance in many biological systems. The discovery of N-glycosylation in bacteria and the functional expression of the N-oligosaccharyltransferase PglB of *Campylobacter jejuni* in *Escherichia coli* enabled the production of engineered glycoproteins and the study of the underlying molecular mechanisms. A particularly promising application for protein glycosylation in recombinant bacteria is the production of potent conjugate vaccines where polysaccharide antigens of pathogenic bacteria are covalently bound to immunogenic carrier proteins.

**Results:**

In this study capsular polysaccharides of the clinically relevant pathogen *Staphylococcus aureus* serotype 5 (CP5) were expressed in *Escherichia coli* and linked *in vivo* to a detoxified version of *Pseudomonas aeruginosa* exotoxin (EPA). We investigated which amino acids of the periplasmic domain of PglB are crucial for the glycosylation reaction using a newly established 96-well screening system enabling the relative quantification of glycoproteins by enzyme-linked immunosorbent assay. A random mutant library was generated by error-prone PCR and screened for inactivating amino acid substitutions. In addition to 15 inactive variants with amino acid changes within the previously known, strictly conserved WWDYG motif of N-oligosaccharyltransferases, 8 inactivating mutations mapped to a flexible loop in close vicinity of the amide nitrogen atom of the acceptor asparagine as revealed in the crystal structure of the homologous enzyme *C. lari* PglB. The importance of the conserved loop residue H479 for glycosylation was confirmed by site directed mutagenesis, while a change to alanine of the adjacent, non-conserved L480 had no effect. In addition, we investigated functional requirements in the so-called MIV motif of bacterial N-oligosaccharyltransferases. Amino acid residues I571 and V575, which had been postulated to interact with the acceptor peptide, were subjected to cassette saturation mutagenesis. With the exception of I571C only hydrophobic residues were found in active variants. Variant I571V performed equally well as the wild type, cysteine at the same position reduced glycoprotein yield slightly, while a change to phenylalanine reduced activity by a factor of three.

**Conclusions:**

This study provides novel structure-function relationships for the periplasmic domain of the *Campylobacter jejuni* N-oligosaccharyltransferase PglB and describes procedures for generating and screening oligosaccharyltransferase mutant libraries in an engineered *E. coli* system.

## Background

N-linked glycosylation is of fundamental importance for the biological activity of many eukaryotic proteins. Bacterial oligosaccharyltransferases (OST) perform a similar type of posttranslational modification in prokaryotic cells and thus hold great promise for producing proteins with defined glycosylation patterns in simple and cost-efficient expression systems such as *Escherichia coli*[1]. The OST of *Campylobacter jejuni* (PglB_*Cj*_) has been functionally expressed in *E. coli*[2] and has been shown to have a relaxed specificity towards its lipid-linked substrate. Therefore it can be exploited, e.g., for the production of conjugate vaccines where polysaccharide antigens of bacterial pathogens are covalently coupled to carrier proteins [3,4]. Polysaccharide-protein conjugates (conjugate vaccines) belong to the most effective measures for combating life-threatening infectious diseases [5]. Diverse O-specific polysaccharides of the outer lipopolysaccharide layer of Gram-negative bacteria can be efficiently coupled to carrier proteins containing a surface-exposed D/E-X_1_-N-*X*_2_-T/S acceptor sequon by PglB_*Cj*_, provided that they contain an N-acetyl sugar at the reducing end [6]. Examples are the O7, O9a, O16 and O157 antigens of *E. coli*, the O1 antigen of *Shigella dysenteriae* as well as the O11 antigen of *Pseudomonas aeruginosa*[3,4,6]. Production of conjugate vaccines by *in vivo* conjugation in *E. coli* is much simpler than with conventional chemical conjugation technologies, and it has already been demonstrated that glycoconjugates can be produced at a larger scale in fed-batch culture with yields of 18–24 mg L^-1^ of purified product [4]. Recently, it was found that capsular polysaccharides of medically important Gram-positive bacterial pathogens such as *Staphylococcus aureus* can also be transferred to acceptor proteins by PglB_*Cj*_ (M. Wacker and J. Lee, in preparation).

PglB_*Cj*_ is a monomeric protein of 713 amino acids and comprises 11 predicted transmembrane helixes in the N-terminal two thirds of the polypeptide and a globular domain at the C-terminus which is soluble and exposed to the periplasm [7]. The latter domain of PglB_*Cj*_ was crystallized by Maita et al. [8]. It harbors the highly conserved W_457_WDYG_461_ motif which is also found in the STT3 subunit of eukaryotic OST [2,8]. When W458 and D459 were both replaced by alanine residues, PglB_*Cj*_ was rendered inactive [2]. The crystal structure of the soluble domain of PglB_*Cj*_ revealed a kinked helix opposite of the WWDYG motif which contained the conserved residues M568, I571 and V575, subsequently termed “MIV” motif of bacterial OST [8,9]. Mutational studies confirmed the importance of I571 for activity [8]. PglB of *Campylobacter lari*, a bacterial OST with 56% amino acid identity to PglB_*Cj*_, also glycosylates recombinant proteins in the periplasm when over-expressed in *E. coli*[10]. The crystal structure of full-length PglB_*C.lari*_ was elucidated recently [11]. The structure contained a divalent metal co-factor as well as a bound synthetic D-Q-N-A-T-*p-*nitro phenylalanine acceptor peptide. It revealed that both the transmembrane and the soluble periplasmic domains of PglB_*C.lari*_ participate in substrate binding and catalysis [11]. The strictly conserved residues W_463_-W_464_-D_465_ of PglB_*C.lari*_ were shown to form a strong network of hydrogen bonds with the hydroxyl group of the threonine residue at position +2 relative to the glycosylated asparagine (N) of the acceptor peptide [11]. This explains the strict requirement for either S or T in the D/E-X_1_-N-*X*_2_-S/T (*X*_2_: any amino acid except proline) consensus sequence of bacterial N-glycosylation sites [12,13]. Furthermore, the crystal structure indicated that conserved residues D56, R147, D154, D156, E319, R331 and R375 of PglB_*C.lari*_ are ligands of the acceptor peptide and/or the metal cofactor. Amino acid changes at D56, D154, E319 of PglB_*C.lari*_ lead to partial or complete loss of activity [11]. The relevance of D54 (equivalent to PglB_*C.lari*_ D56) within the transmembrane domain of PglB_*Cj*_ had been demonstrated previously (by alanine replacement) [8]. A role of the MIV motif in substrate binding is also supported by the PglB_*C.lari*_ structure because the position of the side chain of I572 (equivalent to PglB_*Cj*_ I571) is in agreement with a hydrophobic interaction with the methyl group of the +2 T residue of the acceptor peptide, presumably leading to enhanced affinity for D-X_1_-N-*X*_2_-T acceptor sequons compared to D-X_1_-N-*X*_2_-S as previously shown by Chen et al. [14]. From the full-length structure of PglB_*C.lari*_ it was deduced that binding of acceptor proteins occurs from one side of the protein while undecaprenyl-pyrophosphate-linked oligosaccharide precursors access the active site from the other side [11]. An elongated, flexible loop of the transmembrane domain containing the essential amino acid E319 was suggested to play a central role in catalysis [11]. An *in silico* glycan docking experiment indicated that conserved Y468 (PglB_*Cj*_ Y462) may interact with the N-acetyl group of the innermost sugar of the transferred oligosaccharide [11].

In this study we aimed at elucidating residues of the periplasmic domain of PglB_*Cj*_ which are important for the formation of engineered glycoconjugates. Therefore, we established methodologies for the construction and screening of OST variant libraries in *E. coli*. Two mutant libraries generated by either error-prone PCR mutagenesis of the periplasmic domain or cassette mutagenesis of specific active site residues were analyzed for inactive, but PglB polypeptide producing variants. The obtained results were discussed in the context of the recently published crystal structure of the *C. lari* OST.

## Results

### ELISA detection of glycoconjugates

N-linked glycosylation in recombinant *E. coli* is a powerful method for obtaining conjugates of polysaccharide antigens and immunogenic carrier proteins that can be used as safe and efficient vaccines against bacterial pathogens. A method for detection of glycoproteins in the periplasm of *E.coli* which is suitable for medium throughput screening has not yet been described, but is required for the analysis of *pglB* mutant libraries. Therefore, we developed an enzyme-linked immunosorbent assay (ELISA) in 96-well format and compared the new analytical method to the commonly used Western blot analysis.

**Figure 1:**
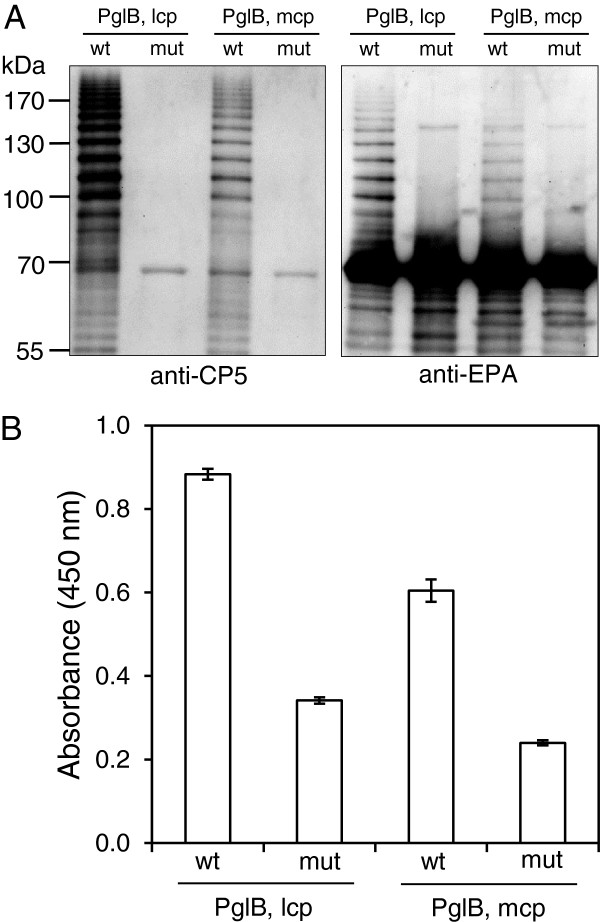
**Western blot and ELISA analysis of periplasmic glycoproteins.** Analysis of periplasmic proteins of EPA-CP5 producing *E. coli* strains and control strains harbouring two different expression vectors for PglB_*Cj*_. (**A**) Western blot of normalised periplasmic samples, detection of glycosylated protein with anti-CP5 (left panel) and anti-EPA antisera (right panel). Theoretical molecular weight of unglycosylated EPA: 67.4 kDa. (**B**) Sandwich ELISA analysis of periplasmic samples for EPA-CP5, average values and standard deviations of ≥ 3 replicates, absorbance measured against air after 15 min development time. lcp: low copy number plasmids pGVXN114 (wt) and pGVXN115 (mut), mcp: medium copy number plasmids pGVXN112Kan (wt) and pGVXN113Kan (mut), mut: inactive PglB W458A-D459A, host strain: *E. coli* CLM24 (pGVXN150, pGVXN345).

In this study we used a plasmid combination facilitating the biosynthesis of conjugates of *Staphylococcus aureus* capsular polysaccharides type 5 (CP5) and the carrier protein *Pseudomonas aeruginosa* exotoxoid A (EPA) (M. Wacker and J. Lee, manuscript in preparation) (Table 1). In strains co-expressing CP5 from plasmid pGVXN345 and EPA with engineered glycosylation sites from plasmid pGVXN150, while at the same time PglB_*Cj*_ was provided from the low copy number plasmid (lcp) pGVXN114, EPA-CP5 glycoconjugates were obtained. A ladder of protein bands reacting with anti-CP5 antibodies was detected in periplasmic extracts of cells grown in shake flask by Western blot analysis (Figure 1A, left panel). Similar protein ladders have been observed previously when O-polysaccharide-protein conjugates were produced in recombinant *E. coli*[3,4]. Random polysaccharide chain lengths result during O-antigen biosynthesis by the combined action of the enzymes Wzy and Wzz [15]. 

**Table 1:** Plasmids used in this study

**Plasmid**	**Description**	**Selection marker**	**Reference**
pACT3	Medium copy number vector for IPTG-inducible expression, *lacI*, *P*_*tac*_, ori: pACYC184	Cm^R^	[25]
pACT3Kan	Kanamycin resistance cassette inserted into chloramphenicol resistance gene of pACT3	Kan^R^	This study
pGVXN114	IPTG-inducible expression of PglB containing a hemagglutinin epitope tag (HA) at the C-terminal end, low copy number plasmid, *lacI*, *P*_*tac*_, ori: IncW	Sp^R^	[4]
pGVXN115	Similar to pGVXN114, but inactive PglB-HA W458A-D459A (PglB mut)	Sp^R^	[4]
pGVXN112Kan	IPTG-inducible expression of PglB containing an HA tag at the C-terminal end, template plasmid for mutant libraries, medium copy number	Kan^R^	This study
pGVXN113Kan	Similar to pGVXN112Kan, but inactive PglB-HA W458A-D459A (PglB mut)	Kan^R^	This study
pGVXN150	L-arabinose inducible expression of periplasmic, hexahistidine-tagged *Pseudomonas aeruginosa* exotoxoid A (EPA) with 2 engineered N-glycosylation sites, *araC*, *P*_*BAD*_, high copy number plasmid, ori: pBR322	Amp^R^	[4]
pGVXN345	Constitutive expression of CP5 capsular polysaccharides of *Staphyloccus aureus*, native promoter, low copy number plasmid, ori: IncPa	Tet^R^, Cm^R^	M. Wacker and J. Lee, in preparation

An alternative medium copy number expression vector for PglB_*Cj*_, pGVXN112Kan (Table 1) also enabled glycosylation of EPA, even though glycoprotein levels were lower (Figure 1A). When periplasmic proteins of control strains harboring the same combinations of EPA and CP5 producing plasmids and expressing mutated, inactive PglB (PglB mut: W458A-D459A) were analyzed, no specific bands were detected (Figure 1A). However, strong bands with the expected size of unglycosylated EPA (MW ≈ 70 kDa) were detected in periplasmic extracts of both wild-type PglB and mutated (mut) PglB strains when blots were analyzed with anti-EPA antibodies (Figure 1A, right panel). A ladder of proteins of a similar size range as the ladder observed in anti-CP5 blots was also seen for wild-type PglB samples on this blot (80–200 kDa). Anti-CP5 specific bands below the size of EPA were most likely due to glycosylated degradation products of EPA. Such degradation products were also observed when engineered EPA was glycosylated with *Shigella* O1 polysaccharides [4].

Next we tested whether glycosylation using this system can also be reliably detected by ELISA, as this method could be used for the analysis of a larger number of clones. The ELISA setup with the best signal-to-noise ratio for periplamic extracts of *E. coli* comprised anti-EPA capturing antibodies and anti-CP5 detection antibodies (sandwich ELISA). ELISA absorbance values were reproducibly and significantly higher for samples from wild-type PglB strains compared to samples from isogenic control strains expressing inactive PglB (Figure 1B). Background signals (PglB mut) were higher when the low copy number *pglB* expression plasmid was used than when the medium copy number, pACT3 derived plasmid was used (Figure 1B). The latter plasmid was used for the construction of *pglB* mutant libraries. In later experiments where mutants were re-analyzed in replicates, a second batch of anti-CP5 antibodies with a higher degree of purity was used, which lead to a reduction of background signals to ± 0.20 (measured against air, i.e., including absorbance of the polystyrene plate and buffer), independent of the type of *pglB* expression plasmid.

The ELISA results for shake flask cultures indicated that the method is suitable for detecting and quantifying glycoproteins in *E. coli* clones expressing different variants of PglB. The coefficient of variation of ELISA absorbance values was below 5% of the mean value for periplasmic extracts of strains expressing either wild-type or inactive PglB. The lower levels of glycoproteins produced by the strain with medium copy number plasmid pGVXN112Kan were reflected in lower values of ELISA absorbance (Figure 1B).

### Screening system for OST mutant libraries

Based on the positive results with the sandwich ELISA assay for shake flask samples, we developed a 96-well cultivation and screening method for glycoprotein production in *E. coli*. Screening of library clones was performed in 96-well format using deep well plates (DWP) for cultivation followed by sucrose-lysozyme extraction of periplasmic proteins and relative quantification of EPA-CP5 glycoconjugates by sandwich ELISA. A test plate was inoculated with 90 clones of the EPA-CP5 producing, wild-type PglB strain (pGVXN112Kan) and with 6 clones of the negative control strain expressing inactive PglB mut (pGVXN113Kan). The ELISA signals of all wells containing the wild-type PglB strain were clearly separated from those wells which contained the inactive control strain (Additional file 1). The average absorbance (coefficient of variation) was 0.402 (5.1%) and 0.275 (4.5%) for wild-type and inactive PglB, respectively. The reproducibility and sensitivity of the DWP-ELISA screening assay was considered sufficiently high for detecting inactivating mutations in *pglB* mutant libraries.

### Random mutagenesis of PglB_*Cj*_ by error-prone PCR

**Figure 2:**
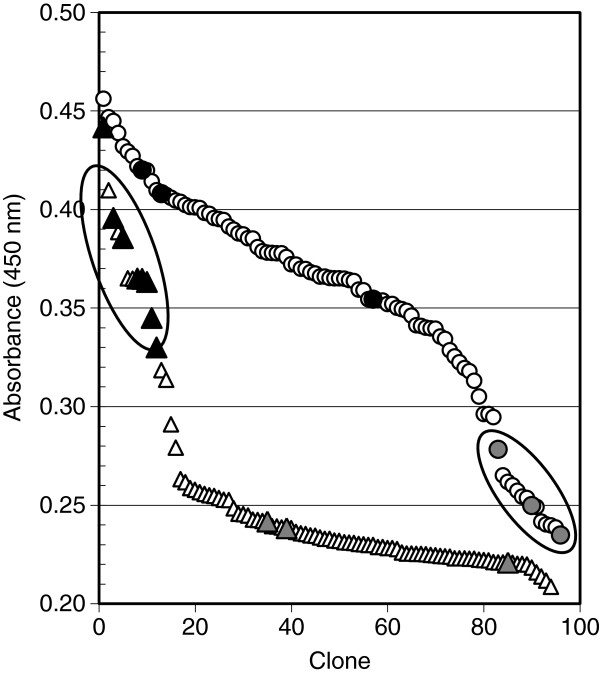
**Screening of OST mutant libraries. Sorted ELISA read-outs of 96-well random mutant libraries of PglB**_***Cj.***_ Open circles: epPCR mutagenesis of the periplasmic domain (H423-I713), inactive clones selected for anti-HA screening and sequencing marked by an elipsoid. Open triangles: Cassette mutagenesis of I571 and V575 using primers with NDT degeneracy, active clones selected for sequencing marked by an elipsoid. Absorbance at 450 nm was measured against air. Filled symbols: wild-type controls on same plate (pGVXN112Kan), shaded symbols: inactive (mut) controls on same plate (pGVXN113Kan), host strain: *E. coli* CLM24 (pGVXN150-pGVXN345).

Procedures for random mutagenesis of *pglB*_*Cj*_ were also developed in the course of this study. The periplasmic domain (H423 to I713) was randomly mutated by error-prone PCR (epPCR) using mutagenic DNA polymerase Mutazyme II. The *pglB* plasmid library was transformed into the expression strain which co-expressed EPA and CP5 polysaccharides. On average 0.8 amino acid changes were introduced within the periplasmic domain of PglB (range 0–2) as shown when 12 randomly picked clones were sequenced. Analysis of 5105 clones with the DWP-ELISA screening system showed that 14.6 ± 4.3% (n = 57 plates) of variants were inactive, i.e. exhibited a background-corrected ELISA signal which was < 20% of the average signal of wt control wells. ELISA data for a representative 96-well epPCR library are shown in Figure 2 (open circles, sorted with respect to detection level). The signals of PglB mut control samples (shaded symbols) always grouped with inactive clones on the right-hand part of sorted ELISA data, while the wild-type controls (black symbols) grouped with active clones (Figure 2). Sequencing of 92 inactive clones without prior analysis of PglB expression showed that 27% of the clones harbored mutations with expected negative effects on expression and/or activity of PglB, i.e., amino acid substitutions within the WWDYG motif (4.4%), mutations to a stop codon (13%), frame shift mutations (4.4%), amino acid substitutions within the MIV motif (1.1%) and changes to potentially structure-disrupting proline residues (4.4%). The overall frequency of clones with one, two and ≥ 3 amino acid changes was 44.6%, 15.2% and 8.3%, respectively. The proportion of wild-type PglB clones was 21.7% while 5.4% harbored silent mutations in the periplasmic domain.

**Figure 3:**
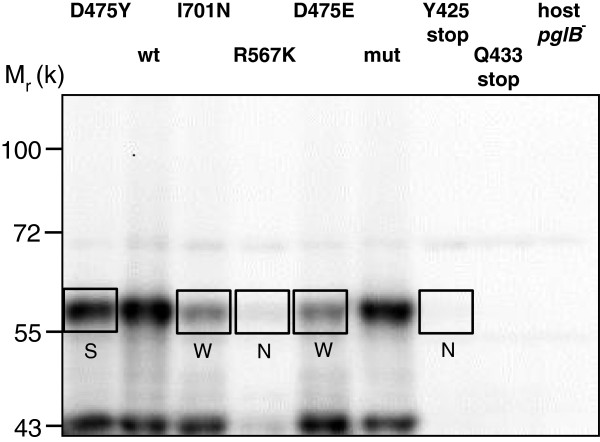
**Analysis of PglB expression by anti-HA Western blot.** Selected inactive clones of epPCR libraries (PglB mutations given in lane header) and control strains were grown and induced in DWP. wt: pGVXN112Kan, mut: pGVXN113Kan, host: *E. coli* CLM24 (pGVXN150, pGVXN345). Relative expression levels of full-length PglB given in subsequent Figures and Tables were determined from the upper, boxed bands by image analysis with the software ImageJ (wt PglB = 100%, S = strong, W = weak, N = negative).

For elucidation of structure-function relationships only inactivating mutations which do not abolish expression of PglB are of interest. The vectors used for expression of *pglB* and construction of mutant libraries yield recombinant protein with a hemagglutinin tag epitope (HA) fused to the C-terminal end (Table 1). Several hundred further epPCR clones with inactive phentotype were screened for production of full-length PglB by anti-HA Western blot analysis. Growth, induction and cell lysis was performed in 96-deep well plates. For validation of the screening method, several clones with one amino acid change were compared to stop codon mutants as well as to wild-type and inactive control strains on one blot. No strong anti-HA specific bands were detected for stop codon mutants and the host strain without *pglB* plasmid (Figure 3). In the case of wild-type PglB and inactive PglB W458A-D459A (mut), two strong bands were detected. Although the position of the upper band was below the expected 83 kDa we assumed this band to represent full-length PglB. Due to specific procedures required for obtaining blots of membrane proteins with sufficient quality, i.e., denaturation at 60°C instead of 95°C, membrane proteins often do not migrate at similar positions as soluble marker proteins in SDS-polyacrylamide gels [16]. For the homologous membrane-bound OST of *C. lari*, which was expressed with a C-terminal HA tag, the anti-HA specific bands on Western blots were also migrating at lower positions than the theoretical molecular mass (between 50 and 75 kDa instead of ≈ 84 kDa, [11]). The lower anti-HA specific bands on our blot may represent the C-terminal, globular domain of PglB generated by limited proteolysis during expression. The theoretical molecular mass of the periplasmic domain including the C-terminal HA tag is 35 kDa. For quantification of relative expression levels of PglB by image analysis only the upper band was used. PglB expression in epPCR variants with one amino acid change ranged from not detectable to wild-type levels (Figure 3). 

When 410 inactive clones of epPCR libraries were screened by anti-HA Western blot analysis, it was found that the majority (68.5%) had lost the ability to produce any PglB, while 84 (20.5%) exhibited weak expression and only 45 (11%) produced PglB at similar levels as the wild-type strain. All clones showing strong PglB-specific bands and 58 clones with weak PglB production were sequenced. The glycosylation deficiency of the PglB positive clones was re-evaluated by DWP-ELISA and could be confirmed in 63% of the cases (ELISA signals < 20% of wt). In the rescreening experiment, the majority of the remaining PglB positive clones exhibited ELISA signals between 20 to 50% of wild-type controls (reduced activity variants, 28.1%) while 8.9% were identified as false negatives (ELISA signals > 50% of wt). Single amino acid substitutions leading to a reproducible lack of glycosylation (ELISA signals < 20% of wt) without abolishing production of PglB are summarized in Table 2. In several cases, the same residues like those hit in single mutants were found to be mutated in PglB-positive clones with two amino acid changes (Table 2, double mutants). Four inactivating amino acid substitutions were identified in two or more individual clones, indicating that the diversity of the epPCR mutant library was covered adequately. The WWDYG motif was hit in 11 single mutant clones and in four clones with two amino acid changes (Table 2). A second region where inactivating mutations clustered was R465 to G481. D475 and G481 were mutated in several clones with strong expression of PglB (Table 2).

**Table 2:** Amino acid substitutions identified in inactive clones

**Single mutants**		**Double mutants**	
**Mutation**	**PglB production**	**Mutation**	**PglB production**
V432F	strong		
W457S	strong	W457R-N582S	weak
W458R	strong		
W458C (3x)	strong		
D459V (3x)	strong	D459V-A448T	weak
		D459N-S492C	strong
Y460N	strong		
Y460C	weak		
G461D	strong	G461D-S488P	weak
R465H	strong	R465H-G477V	weak
D469L	strong		
D475F	strong		
D475Y	strong		
D475E	weak		
G476D	weak	G476D-K646I	weak
G481D	strong		
G481N	strong		
G481S (2x)	strong		
S488P (2x)	weak	S488P-V631I	weak
D494V	weak		
M501K	weak		
A528V	strong		
K556N	weak		
A566S	weak		
A682D	strong		

Random mutagenesis of *pglB*_*Cj*_ by epPCR was restricted to the region encoding the periplasmic domain (H423-I713). All clones reproducibly exhibited ELISA signals for EPA-CP5 which were ≤ 20% of the wild-type PglB control strain (pGVXN112Kan). Only results for clones which expressed detectable amounts of PglB in DWP are shown (analysed by anti-HA Western blot).

**Figure 4:**
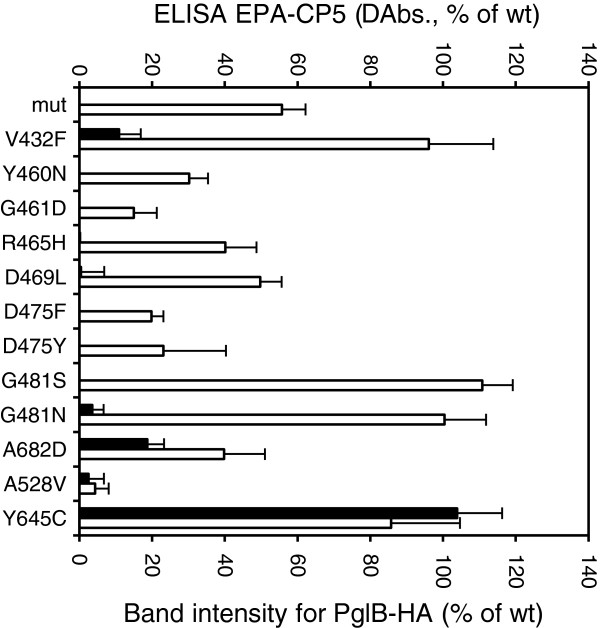
**Relative levels of glycoprotein and PglB expression in epPCR variants.** EPA-CP5 levels in periplasmic extracts of deep well plate cultures were determined by sandwich ELISA (solid bars, left y-axis, wt = 100%). ELISA signals were corrected for absorbance of PglB mut control wells (ΔAbs. 450 nm). PglB production was analysed by anti-HA Western blot of total cell protein samples (open bars, right y-axis, band intensity of wt = 100%). Average values and standard deviations of three replicate deep well cultures.

Selected variant plasmids were isolated by mini-prep, re-transformed into the expression strain *E. coli* CLM24 (pGVXN150, pGVXN345) and re-analyzed in triplicate with the DPW-ELISA and anti-HA screening systems. Mutation V432F reduced background-corrected EPA-CP5 signals by 89% without any negative effect on expression of PglB (Figure 4). Change of G481 to either S or N abolished formation of glycoconjugates completely in spite of wild-type levels of PglB detected by anti-HA Western blot (Figure 4). In addition to mutations Y460N and G461D within the WWDYG motif, four other amino acid changes lead to a complete loss of glycosylation activity: R465H, D469L, D475F and D475Y. Lack of glycosylation in these cases cannot be explained solely by reduced expression of the OST. There were still substantial amounts of PglB-HA detectable by anti HA Western blot (20-50% of wt levels, Figure 4). In the case of variants A682D and A528V, the strongly reduced yields of EPA-CP5 are most likely related to a concomitant reduction in PglB expression levels (Figure 4). As a control, an epPCR variant with an amino acid substitution with no effect, Y645C, was also analyzed within the same DWP-ELISA assay. For this clone, no significant difference in ELISA signals and expression levels of PglB to the wild-type control was observed (Figure 4). Y645 resides in a non-conserved region of PglB distal to the active site.

**Figure 5:**
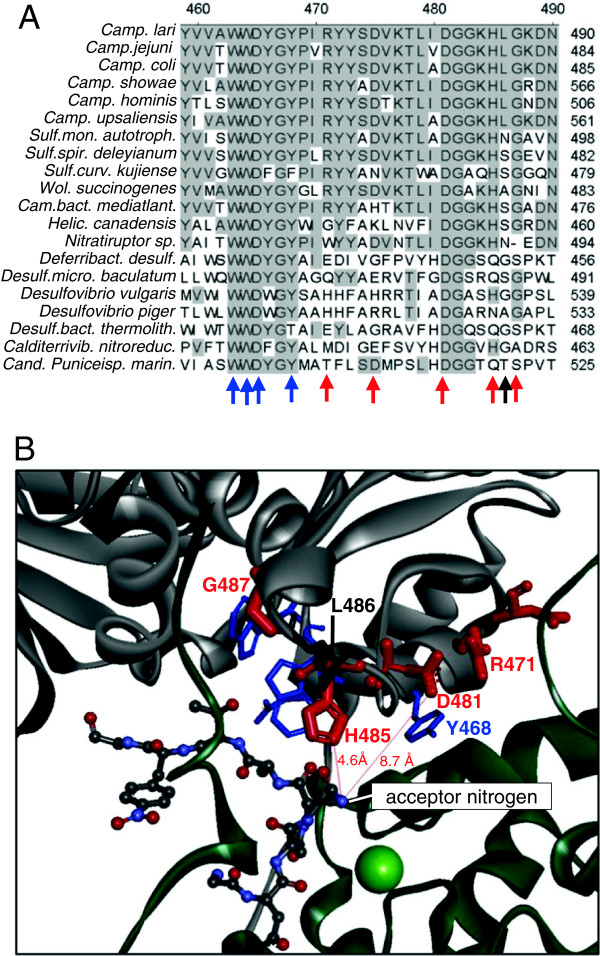
**Structural context of inactivating mutations close to the WWDYG motif.** (**A**) Alignment of bacterial PglB sequences in the region covering the *C. jejuni* W_457_WDYG_461_ motif to *C. jejuni* G481. Numbering of ruler according to PglB_*C. lari*_, conserved residues shaded, residues shown in B marked by arrows. (**B**) Position of activity-relevant residues which were identified by epPCR of PglB_*Cj*_ in the crystal structure 3RCE of PglB_*C. lari*_ (bold red) [11]. The WWD residues and conserved Y468 are shown in blue. Non-conserved, mutation-tolerant residue L486 (*C. jejuni* L480) is shown in black. Backbone structures of the periplasmic domain and membrane domains are shown in grey and dark green, respectively. The bound bound D-Q-N-A-T-*p*-nitrophenylalanine acceptor peptide is given as atom-coloured ball-stick representation (N = position 0) and the coordinated Mg^2+^ ion is shown in light green.

D475 and G481 are highly conserved in bacterial PglB homologues (Figure 5A). In the crystal structure of PglB_*C. lari*_ the corresponding amino acids (D481 and G487) are part of a flexible loop which is localized just above the amide nitrogen atom of the acceptor peptide where the covalent linkage to the reducing sugar of the undecyprenyl-linked oligosaccharide substrate occurs (Figure 5B). To further confirm the importance of this loop we changed the conserved residue H479 (H485 in *C. lari*), which is located in only 4.6 Å distance to the acceptor nitrogen atom (Figure 5B), to glutamine, an amino acid which occurs in several distantly related PglB_*Cj*_ homologues (Figure 5A). For comparison, the adjacent, non-conserved residue L480 (L486 in *C. lari*) with an upward orientation of the side chain in the *C. lari* PglB crystal structure was mutated to alanine (Figures 5A + B). The PglB H479Q variant was expressed at wild-type levels, with anti-HA band intensities reaching 97 ± 23% of wild-type PglB (n = 3). However, the yield of glycoprotein was reduced more than five-fold as determined by sandwich ELISA (16 ± 2.6% of background-corrected wild-type signals, n = 3). By contrast, we did not observe any significant difference between variant L480A and wild-type PglB_*Cj*_ in EPA-CP5 and PglB levels (band intensities on anti-HA Western blots: 127 ± 28% of wild-type, background-corrected ELISA signals: 118 ± 0.04% of wild-type, n = 3). In agreement with its relevance for activity, H479 was also found to be mutated in an inactive, PglB-positive epPCR clone with three amino acid changes (H479R-D589E-F679I).

The epPCR experiment did not yield any variants with reproducibly increased glycosylation efficiency; however, numerous variants with neutral single or double mutations were found, e.g., V470M, D705Y and K603E-S664R all of which exhibited EPA-CP5 ELISA signals similar to wild-type PglB. In contrast to residues mutated in inactive variants, only non-conserved residues were mutated in the latter cases.

### Cassette mutagenesis of I571 and V575 (MIV motif)

**Figure 6:**
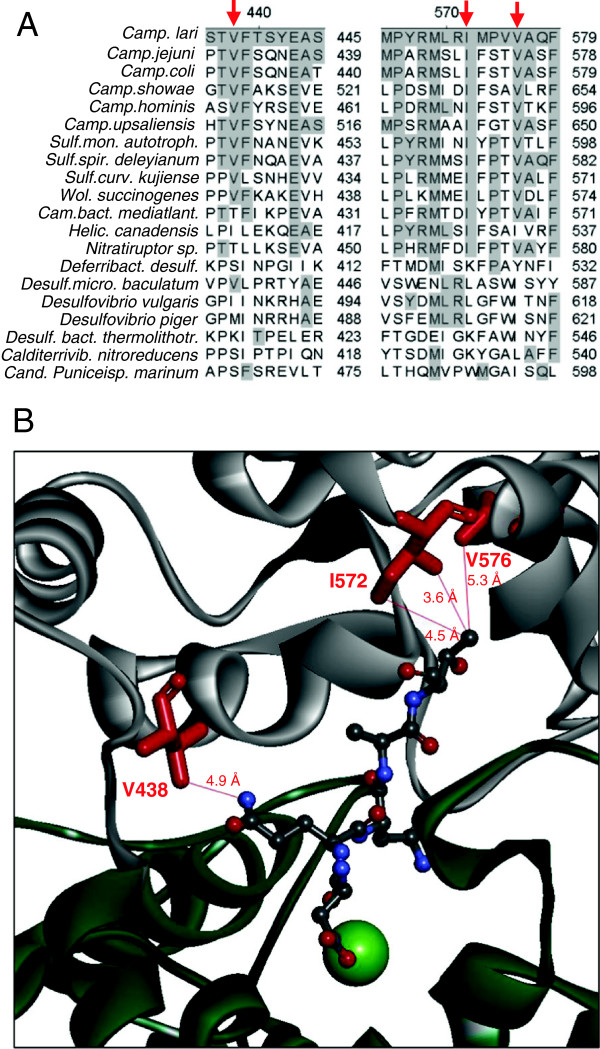
**Structural context of V432 and the MIV motif.** (**A**) Alignment of bacterial PglB sequences in the vicinity of *C. jejuni* V432 and I571/V575. Numbering of ruler according to PglB_*C. lari*_, conserved residues shaded, residues shown in B marked by arrows. (**B**) Position of residues equivalent to PglB_*Cj*_ V432, I571 and V575 in the crystal structure 3RCE of PglB_*C.lari*_[11] (red). Periplasmic domain in grey, transmembrane domain in dark green, bound D-Q-N-A-T- *p*-nitrophenylalanine acceptor peptide as atom-coloured ball-stick representation, Mg^2+^ ion as light-green sphere.

Opposite of the strictly conserved WWDYG motif of N-oligosaccharyltransferases, three conserved hydrophobic residues are found in bacterial enzymes which comprise the so-called “MIV” motif [8] (amino acids M568, I571 and V575 in PglB_*Cj*_, corresponding to M569, I572 and V576 in *C. lari*, Figure 6A). M568 was hit once in the epPCR experiment, but no inactivating mutations were recovered for I571 or V575. In order to elucidate the plasticity and structural requirements at these sites, random cassette mutagenesis was performed. Randomization was achieved with mutagenic primers bearing NDT degenerate codons (N = A, T, G or C; D = A, T or G) instead of wild-type ATT and GTG at positions I571 and V575, respectively. NDT mutagenesis introduces codons for L, I, V, F, Y, G, S, C, D, N, H, and R at targeted positions in gene sequences and combines a high degree of chemical diversity with a relatively small library size [17]. After confirming the success of NDT mutagenesis by sequencing of random clones, the *pglB* plasmid library was re-transformed in the expression strain *E. coli* CLM24 (pGVXN150, pGVXN345) and screened with the DWP-ELISA system. Sorted ELISA data for a representative 96-well library are shown in Figure 2 (open triangles). None of the library clones exhibited increased EPA-CP5 ELISA signals compared to the top signal from wild-type clones of the same 96-well screening plate. The majority of clones was inactive (77 ± 8% of samples with an ELISA absorbance below 10% of wt, n = 6 96-well plates). Only 12 out of 590 screened library clones produced signals within the range of wild-type control clones (e.g., clones marked in Figure 2). Three of the active clones corresponded to the quick change template and two clones carried silent mutations introduced with the NDT primers, i.e. control mutation F572: TTT > TTC and V575: GTG > GTT due to the NDT degenerate codon. In the other active clones several single and double amino acid substitutions were identified which are listed in Table 3. One particular substitution, I571V, was found in three clones with one amino acid change and in two clones with two changes. With the exception of I571C, hydrophobic side chains were replaced by other hydrophobic side chains in active variants (Table 3). In comparison to amino acids bearing hydroxyl, amine, amide or carboxyl functional groups in their side chains, cystein is non-polar/neutral. 

**Table 3:** Mutations tolerated at the MIV motif

**PglB variant**	**ELISA EPA-CP5**		**PglB polypeptide**
	Abs. 450 nm	% of wt	anti-HA, % of wt
wt	0.57 (±0.03)	100	100 (±13)
mut	0.20 (±0.01)	0	56 (±7)
I571V	0.51 (±0.05)	85	89 (±28)
I571V V575I	0.52 (±0.04)	87	85 (±11)
I571V V575L	0.46 (±0.04)*	70	84 (±7)
I571F V575L	0.32 (±0.01)*	34	96 (±31)
I571C V575I	0.41 (±0.03)*	58	65 (±13)

ELISA absorbance measured against air. Average values and standard deviations of at least n = 3 replicate mini-well cultures.

* Significant difference to wt and mut, *P* value of Student’s *T*-test < 0.001.

The relative levels of EPA-CP5 and PglB were analyzed for each type of PglB variant in replicate DWP mini-cultures after re-transformation of the sequenced variant plasmids (Table 3). No significant difference to wild-type PglB was found for variants I571V and I571V-V575I, respectively (Table 3). In the case of I571V -V575L, a moderate, but significant reduction of EPA-CP5 biosynthesis was observed which was not due to reduced expression of PglB (Table 3). The level of EPA-CP5 detected by ELISA was reduced by a factor of three in variant I571F-V575L without any reduction of PglB expression, indicating that an F at position 571 has a negative effect on OST activity (Table 3). The double mutant I571C-V575I was characterized by a parallel, moderate reduction in the levels of both EPA-CP5 and PglB (Table 3). The alignment of a set of diverse, non-redundant amino acid sequences of bacterial PglB homologues indicates that I571 and V575 are strictly conserved in ε-Proteobacteria, but not in other species (Figure 6A). However, also in non-ε-Proteobacteria mostly hydrophobic amino acids occur at the corresponding positions (Figure 6A). Isoleucine and leucine at position PglB_*Cj*_ V575 were found in active variants of the cassette mutagenesis experiment and also occur in natural PglB sequences (Figure 6A). The spatial orientation of the equivalent residues I572 and V576 of PglB_*C. lari*_ towards the bound acceptor peptide is shown in Figure 6B. The aliphatic side chains of both amino acids are in close proximity to the methyl group of the threonine residue at the N + 2 position of the D-Q-N-A-T-*p*-nitro phenylalanine acceptor peptide (Figure 6B).

## Discussion

The use of bacterial OST in recombinant *E. coli* facilitates *in vivo* production of conjugates of polysaccharide antigens of pathogenic bacteria and immunogenic carrier proteins [3,4,18]. Recombinant *E. coli* equipped with the oligosaccharyltransferase PglB of *C. jejuni* is the most advanced system for the biotechnological production of N-linked conjugate vaccines.

In order to gain a better understanding of residues of PglB_*Cj*_ required for the glycosylation reaction we studied the effect of random mutations on the formation of glycoconjugates composed of *Pseudomonas aeruginosa* exotoxoid and *Staphylococcus aureus* capsular polysaccharide CP5. Therefore, we established methods for constructing epPCR mutant libraries of recombinant PglB and for the screening of glycoprotein-producing *E. coli* in 96-well format. Growth and induction of *pglB* library clones in deep well plates followed by sandwich ELISA analysis of the periplasmic extracts allowed a reliable distinction of active and inactive OST variants.

The screening of an epPCR library where the periplasmic domain of PglB_*Cj*_ had been mutagenized facilitated the identification of previously unknown residues which are relevant for OST activity. Apart from confirming the importance of the well-known, strictly conserved W_457_WDYG_461_ residues of PglB_*Cj*_, the epPCR experiment revealed that the region V464 to G481 immediately adjacent to this motif is also highly important (Table 2). In the partial crystal structure of PglB_*Cj*_ and the recently revealed full-length structure of PglB_*C.lari*_, these amino acids form a flexible loop in close proximity to the side chains of tryptophan and aspartic acid residues of the WWDYG motif [8,11]. Due to the spatial orientation of the bound acceptor peptide and the assumed reaction mechanism of PglB [11], this loop is believed to interact with sugar moieties of the glycan substrate. The highly conserved residue G481, which was hit several times in inactive epPCR mutants, could function as a helix breaker before the ascending helix starting at K482, thereby providing the loop D475-G481 (*C. lari* D481-G487) with sufficient flexibility for interaction with the oligo-/polysaccharide (Figure 5B). The inactivating mutations at G481 identified in our epPCR experiment presumably induce structural changes which disturb the interaction of loop residues such as H479 and D475 with the glycan substrate. In most homologous sequences which do not bear a glycine at this position, a helix-breaking glycine or proline residue is present at an adjacent position (Figure 5A). Our results further confirm the importance of the strictly conserved aspartic acid residue at position 475 (*C. lari* D481) for activity of PglB_*Cj*_ as proposed by Maita et al. [8]. In a study using radio-labeled undecaprenyl-pyrophosphate linked oligosacharides of *C. jejuni* at limiting concentrations and a synthetic Ac-D-Q-N-A-T-*p*-nitro phenylalanine acceptor peptide in excess, mutations D475E and D475A decreased glycosylation rates of PglB_*Cj*_ by a factor of four and six, respectively [19]. The negative effect of the mutations was strongest at low glycan substrate concentrations [19]. Together with the reduced activity of a K478A mutant reported by the same authors these results support the importance of the flexible loop D475-G481 and its probable involvement in glycan binding as suggested by our data. Judged from its distance to the acceptor nitrogen atom (Figure 5B), the carboxylate group of D475 might be involved in a hydrogen bond network coordinating the C2 N-acetyl group of the innermost sugar of the transferred glycan. Similarly to eukaryotic OST, PglB_*Cj*_ is believed to require an N-acetyl sugar at the reducing end for efficient transfer of poly- and oligosaccharides to acceptor proteins [6]. The side chain of the strictly conserved tyrosine residue at position 462 (*C. lari* Y468) is in close proximity to D475 (*C. lari* D481) (Figure 5B). For *C. lari* Y468 an interaction with the N-acetyl group of the innermost sugar has been suggested by others based on *in silico* glycan docking [11]. The importance of the loop D475-G481 is further backed by the phenotype of the H479Q variant generated in this study. The side chain of the equivalent residue H485 in PglB_*C.lari*_ is spatially very close to the nucleophilic amide nitrogen of the bound D-Q-N-A-T-*p*-nitro phenylalanine acceptor peptide (Figure 5B). With few exceptions glutamine is the only other amino acid occurring at this position in bacterial PglB homologues (Figure 5A). The side chain of glutamine has a similar length as that of histidine and also contains an amino functionality. In PglB_*Cj*_ glutamine seems to be able to substitute for the function of histidine at position 479 only to a limited degree. Interestingly, removal of the aliphatic side chain of the adjacent, non-conserved residue L481 by mutation to alanine had no effect on activity at all. This is in agreement with the spatial orientation of the side chain of the equivalent residue L486 in PglB_*C.lari*_ pointing away from the acceptor peptide (Figure 5B).

In only one inactive clone with strong expression of *pglB* an amino acid change was found which mapped to a region outside of W457 to G481. The mutation V432F strongly reduced the glycoprotein specific ELISA signals, but the variant was not completely inactive as EPA-CP5 formation was still detectable. Judged from the position of the equivalent residue V438 in the crystal structure of PglB_*C.lari*_, the bulkier and less flexible phenyl side chain in the variant enzyme might interfere with the binding of D-X_1_-N-*X*_2_-T/S acceptor peptides. In particular, steric hindrance of the side chain of the glutamine residue at the −1 position in D-Q-N-X-T/S sequons could play a role (Figure 6B). In the engineered EPA used in this study as acceptor protein two glycosylation sites had been introduced in surface exposed loops, D-N-N-N-S and D-Q-N-R-T. It would be interesting to study whether glycosylation of the D-Q-N-R-T site is more affected than that of the other site and whether a change of −1 Q to amino acids with shorter side chains could compensate for the negative effect of PglB_*Cj*_ V432F. V432 is not strictly conserved in PglB homologues; however, in ε-Proteobacteria a short “VF” motif is present at this position in most species (Figure 6A).

The so-called MIV motif of bacterial N-oligosaccharyltransferases (M568, I571, V575 in PglB_*Cj*_, Figure 6A) which is assumed to interact with the acceptor peptide was hit only once in the epPCR experiment. The side chains of the equivalent residues I572 and V576 of PglB_*C.lari*_ are very close to the side chains of the WWDYG motif in the crystal structure (Figure 6B). Therefore, we specifically randomized these residues in PglB_*Cj*_ using degenerate primers and screened the resulting focused library. Our results indicate that only few other, chemically similar amino acids are tolerated at these positions. Wild-type isoleucine and valine at position 571 exhibited the same phenotype. Presumably, both side chains allow for sufficient hydrophobic interaction with the methyl group of +2 T of the acceptor sequon as suggested for PglB_*C.lari*_[11]. The strong preference for hydrophobic residues at I571 as well as V575 in active variants indicates that a hydrophobic surface promotes binding of the polypeptide chain of the acceptor protein. In addition to I572 and V576, numerous other surface-exposed amino acids of the acceptor peptide binding pocket are also hydrophobic in PglB_*C.lari*_: I317, M318, Y433, V438, Y466, Y468 and V575 [11]. With the exception of M318 and V575 these residues are conserved in PglB_*Cj*_ (data not shown). An elongated hydrophobic patch containing the WWDYG motif in its center was suggested to interact with acceptor polypeptides in the archaeal N-oligosaccharyltransferase of *Pyrococcus furiosus*[20].

Directed evolution, comprising multiple rounds of random mutagenesis, randomization of active site residues and recombination of beneficial mutations, has proven to be a powerful method for tuning enzymes to specific needs, provided that suitable screening systems are available [21-23]. Theoretically, it should be possible to evolve bacterial OSTs for enhanced activity towards non-native polysaccharide substrates, although no such studies have yet been reported in the literature. We did not detect any PglB variants with increased yields of glycoprotein in our libraries. Most likely, the number of screened epPCR clones was too low to find improved variants, which are expected to be very rare. Amino acid substitutions at the MIV motif were at best neutral, which indicates that it is difficult to further optimize interactions of D-X_1_-N-*X*_2_-S/T acceptor peptides with PglB. However, we cannot exclude the possibility that protein glycosylation in the applied *E. coli* system was limited by other factors than OST activity, e.g., biosynthesis of undecaprenyl-pyrophosphate linked polysaccharide precursors. Furthermore, the crystal structure of full-length PglB_*C.lari*_ strongly suggests that residues of the membrane domain are also involved in the catalytic mechanism [11]. It might well be that an increase in catalytic activity is not possible without simultaneous changes both in the periplasmic and membrane domains of PglB_*Cj*._

A general problem of enzyme engineering is the destabilizing effect of introduced mutations. In the case of PglB_*Cj*_ this was reflected in a high proportion of amino acid substitutions having detrimental effects on production and stable maintenance of the PglB polypeptide. As conserved positions of PglB_*Cj*_ such as I571 and V575 were found to be particularly sensitive to changes, future mutagenesis strategies should focus on non-conserved residues in the vicinity of the active site. In addition, randomization at specific positions might be restricted to amino acids found in natural sequences as proposed by Jochens and Bornscheuer [24], thereby reducing the number of unwanted instable variants and thus the screening effort.

## Conclusions

In summary, this study adds important information to structure-function relationships of bacterial OST and provides methods for generating and screening OST mutant libraries in *E. coli* strains capable of producing engineered glycoproteins.

## Methods

### Bacterial strains and plasmids

*Escherichia coli* CLM24 (O polysaccharide ligase WaaL negative derivative of W3110, [3]) was used as host strain for all *in vivo* glycosylation experiments. For construction of plasmid libraries by cassette mutagenesis, *E. coli* XL10 Gold cells were used (Agilent-Stratagene, La Jolla, CA, USA).

Plasmids used in this study are listed in Table 1. Expression vector pACT3Kan was constructed by insertion of a Kanamycin resistance cassette into the *cat* chloramphenicol resistance gene of pACT3 [25] by homologous recombination. Plasmids pGVXN112Kan and pGVXN113Kan were constructed by PCR-sub cloning of the sequence coding for wt PglB with C terminal HA tag and inactive PglB-HA W458A-D459A from pGVXN114 and pGVXN115, respectively, into pACT3Kan using restriction sites *Kpn*I and *Bam*HI. Construction of plasmid pGVXN345 for expression of the CP5 polysaccharide biosynthesis cluster of *S. aureus* will be described elsewhere (GlycoVaxyn and Jean Lee, manuscript in preparation).

### Expression of N-glycoproteins in shake flasks

Glycoprotein producing and control strains were cultivated in 100 mL Erlenmeyer flasks with 70 mL LB medium (5 g L^-1^ yeast extract, 10 g L^-1^ casein-based tryptone, 5 g L^-1^ NaCl) supplemented with 100 mg L^-1^ ampicillin, 30 mg L^-1^ kanamycin and 20 mg L^-1^ tetracycline. Flasks were inoculated 1:100 from overnight tube cultures and incubated at 37°C and 160 rpm. When an OD_600_ of ≈ 0.5 was reached 1 mM IPTG and 2 g L^-1^ L-arabinose were added and 4 h later again 2 g L^-1^ L-arabinose. Cells were harvested after overnight incubation by centrifugation (10’000 x g, 15 min, 4°C). Periplasmic proteins were extracted by sucrose-lysozyme treatment [3]. Cell pellets were resuspended to an OD_600_ of 20 in lysis buffer containing 20% w/v sucrose, 30 mM Tris·HCl (pH 8.0), 1 mM EDTA, 1 mg mL^-1^ lysozyme, 1 tablet per 80 mL of Complete^TM^ protease inhibitor mix (Roche, Basel, Switzerland) and incubated for 1 h on ice with gentle magnetic stirring. Spheroblasts with cytoplasmic and membrane proteins were separated by centrifugation (5000 x g, 15 min, 4°C) and the supernatant was used for ELISA and Western blot analysis.

### Western blot analysis

Glycoproteins and oligosaccharyltransferase expressed by recombinant *E. coli* were analyzed by SDS-PAGE and subsequent Western blot following standard electrophoresis, blotting, immunoreaction and washing procedures. For analysis of EPA-CP5, extracts of periplasmic proteins were supplemented with similar volumes of 2 x SDS PAGE sample buffer, denatured for 5 min at 95°C and similar volumes were loaded on acrylamide gels. EPA and EPA-CP5 glycoproteins were detected with anti-EPA (rabbit, Sigma-Aldrich, Buchs, Switzerland) and anti-CP5 antibodies (rabbit, GlycoVaxyn AG), respectively.

For Western blot analysis of membrane bound PglB-HA, total cell protein samples were prepared by resuspending cell pellets obtained from overnight induced 1 mL mini-cultures in deep-well plates (see below) in 100 μL of 1 x SDS-PAGE sample buffer followed by denaturation at 60°C for 30 min. In order to facilitate relative quantification, similar volumes of protein samples were loaded per well (5 μL). PglB-HA protein bands were visualized by hybridization with anti-HA antibodies (rabbit, Sigma-Aldrich).

Western blots were generally developed by hybridization with secondary anti-rabbit IgG-HRP antibodies (goat, Bio-Rad) followed by reaction with Super Signal Dura West HRP substrate according to the manufacturer’s instructions (Thermo-Scientific-Pierce, Rockford, IL, USA). Chemiluminescence signals were recorded with a ChemiDoc-It imaging system (UVP, Upland, CA, USA). Relative intensities of bands specific for full-length PglB-HA (upper bands in Figure 3) were quantified by using band area and relative Gray values calculated with the image analysis software ImageJ (http://rsbweb.nih.gov/ij/).

### Enzyme linked immunosorbent assay (ELISA)

The relative amount of glycosylated proteins in different periplasmic extract samples was analyzed by sandwich ELISA using transparent polystyrol 96-well plates with high capacity for protein binding (F96 MaxiSorp, Nunc). For coating with the capture antibody, wells were filled with 60 μL of protein G purified goat-anti-EPA antiserum (diluted in PBS to ≈ 10 μg mL^-1^) (US Biological/Lucerna Chem AG, Lucerne, Switzerland) and incubated overnight at 4°C without shaking. After four washing steps of 2 min with 300 μL PBS containing 0.05% v/v Tween-20, wells were blocked for at least 2 h at room temperature with 300 μL PBS containing 10% w/v dry milk. Following another washing step of 2 min with 300 μL PBS-Tween, 50 μL of periplasmic extract was added per well. For hybridization of the analyte with the capture antibodies, micro plates were incubated overnight at 4°C. Unbound EPA/EPA-CP5 was removed by washing the plate four times for 2 min with 300 μL PBS-Tween per well. Secondary antibodies (rabbit anti-CP5 antiserum, GlycoVaxyn AG) were added as 1:100 dilution in PBS + 1% dry milk (50 μL per well), followed by incubation for at least 1 h at room temperature and 10 rpm. Excess antibodies were removed by four washing steps as described above. Detection antibodies (goat-anti-rabbit IgG-HRP, Bio-Rad, Reinach, Switzerland) were added as 1:20’000 dilution in PBS + 1% dry milk, followed by incubation for at least 1 h (room temperature, 10 rpm) and four final washing steps. Light-protected ELISA plates were developed with Ultra-TMB-ELISA HRP substrate (Thermo-Scientific/Pierce) according to the manufacturer’s instructions. ELISA signals were recorded at 450 nm using a Cary 50 MPR microplate reader.

### Random mutagenesis methods

Random mutations were introduced into the *pglB* gene by error-prone PCR (epPCR) using mutagenic Mutazyme II DNA polymerase (Agilent-Stratagene). Due to the very low transformation effiency of spectinomycin-selectable, low copy number expression vector pGVXN114, an alternative, kanamycin-selectable, medium copy number expression vector (pGVXN112Kan) was used as template for the construction of *pglB* mutant libraries. The soluble, periplasmic domain of PglB (residues H423 to I713) was amplified in 25 PCR cycles using forward primer 5’-GCAACTATTTTGACTTTAGCTCCAGTATTTATC-3’ and reverse primer 5’-CTAGAGGATCCTTAAGCGTAATCTGGAACATCGTATGGGTA-3’. The concentration of the template plasmid was 17 ng μL^-1^ and the annealing temperature was set to 55°C. PCR products with the expected a size of ≈ 900 bp were purified with a gel extraction kit (Sigma, Buchs, Switzerland). Purified epPCR product was used in 50-fold molar excess as mega primer in a quick change-like PCR reaction (25 cycles) using high fidelity DNA polymerase (Phusion^TM^, New England Biolabs, Ipswich, MA, USA) and 1 ng μL^-1^ of pGVXN112Kan as template. Methylated wt plasmid DNA was removed by *Dpn*I digest. Salts and reagents were removed with a PCR clean-up kit (elution with ddH_2_O). Electro competent *Escherichia coli* CLM24 (pGVXN150, pGVXN345) were prepared by standard procedures and transformed with 2 μL of purified mega primer-quick change product using electroporation (1 mm gap width cuvettes, 5 ms pulses of 1.8 kV). Transformants were selected on LB Agar supplemented with 100 mg L^-1^ ampicillin, 30 mg L^-1^ kanamycin and 20 mg L^-1^ tetracycline.

Cassette mutagenesis of I571 and V575 was performed by performing quick change PCR with Phusion^TM^ polymerase (New England Biolabs). Wild-type plasmid DNA (pGVXN112Kan, 1 ng μL^-1^) was amplified with mutagenic primers in 18 cycles, the annealing temperature was 55°C. The sequences of forward and reverse primers were 5'-T TAT ATG CCC GCT AGA ATG TCT TTG NDT TTC TCT ACG NDT GCT AGT TTT TCT TTT TAT TAA TTT AG-3' and 5'-CT AAA TTA ATA AAA AGA AAA ACT AGC AHN CGT AGA GAA AHN CAA AGA CAT TCT AGC GGG CAT ATA A-3', respectively (mutagenized codons and silent control mutation TTT > TTC underlined). After *Dpn*I digestion the PCR product was directly used for heat shock transformation of commercial ultra-competent *E. coli* XL10 Gold (Agilent-Stratagene). Transformants were selected on LB-Kanamycin plates. After confirming the quality of the library by sequencing of randomly picked XL10 Gold clones, several thousand XL10 Gold colonies were resuspended in phosphate buffered saline (PBS) and mixed. The plasmid library was isolated with a mini-prep kit and transformed into the expression strain *E. coli* CLM24 (pGVXN150, pGVXN345).

### Screening system

For mutant screening, individual *E. coli* clones were cultivated in 96-deep well plates (DWP) with a total volume of 2.2 mL per well (riplateTM sw 2 mL, Ritter GmbH, Schwabmünchen, Germany). For overnight pre-cultures, wells were filled with 0.5 mL of buffered medium containing 5 g L^-1^ yeast extract, 10 g L^-1^ tryptone, 12.8 g L^-1^ Na_2_HPO_4_·7H_2_O, 3.0 g L^-1^ KH_2_PO_4_, 0.5 g L^-1^ NaCl, 1.0 g L^-1^ NH_4_Cl, 2 mM MgSO_4_·7H_2_O and 0.1 mM CaCl_2_, 100 mg L^-1^ ampicillin, 30 mg L^-1^ kanamycin and 20 mg L^-1^ tetracycline.

DWPs were sealed with perforated rubber-metal lids with thin sheets of PTFE and microfiber filter material as sterility barrier (SMCR1296 sandwich cover, system Duetz, Kuhner AG, Birsfelden, Switzerland). Preculture plates were inoculated with single colonies from library transformation plates and incubated at 37°C and 500 rpm for 16–20 h in a microplate shaker (Infors AG, Bottmingen, Switzerland). Aliquots of pre-culture plates (100 μL) were transferred to sterile 96-well microtiter plates (Nunc, Roskilde, Denmark), supplemented with an equal volume of medium containing 50% v/v glycerol and stored at −80°C.

Expression DWPs contained 1.3 mL of the medium described above per well and were inoculated 1:50 from pre-culture plates. Plates were incubated at 37°C and 500 rpm. OD_600_ was determined after 4–5 h and then every hour with a microplate reader (Cary 50 MPR, Varian, Palo Alto, CA, USA). At an OD_600_ of ≥ 0.4 expression of PglB and carrier protein was induced by adding 1 mM IPTG and 4 g L^-1^ L-arabinose, respectively, and agitation was reduced to 100 rpm. After overnight incubation (induction time 15–20 h) 1 mL of culture fluid per well was transferred to a new DWP and cells were separated by centrifugation (4000 rpm, 15 min, 4°C). Cell pellets in wells were resuspended in 200 μL ice-cold lysozyme-sucrose lysis buffer (see above). DWPs were incubated without agitation for 1 h at 4°C and cell suspensions were then transferred to a transparent microtiter plate and centrifuged at 3000 rpm and 4°C for 15 min. The supernatant (extract of periplasmic proteins) was used for ELISA analysis in 96-well plates (see above).

### DNA and protein sequence analysis

For analysis of library quality, plasmid mini-preps of random clones of epPCR and cassette mutagenesis experiments were sequenced using primers binding within the second half of *pglB* (sequencing service: GATC Biotech GmbH, Constance, Germany). Inactive clones with positive response on PglB blots were collected in new 96-well glycerol stock libraries and sequenced using automated 96-well plasmid extraction (GATC Biotech). DNA and protein sequences were compared to the wild-type sequence using Lasergene^TM^ Seqbuilder^TM^ and MegAlign^TM^ programs (DNASTAR, Madison, WI, USA). Mutations were manually checked for lack of ambiguity in sequencing chromatograms. Non-redundant protein sequences homologous to PglB_*Cj*_ were retrieved from a protein BLAST search on the NCBI website and aligned using the ClustalW algorithm of MegAlign^TM^. For visualisation of individual residues and spatial distances in the 3D-structure of PglB, the crystal structure file 3RCE [11] was retrieved from Protein Data Bank and displayed using the program Discovery Studio Visualizer 3.1 (Accelrys, Cambridge, UK).

## Abbreviations

CP5: *Staphylococcus aureus* capsular polysaccharide serotype 5; EPA: *Pseudomonas aeruginusa* exotoxin (toxoid form); DWP: deep well plate; ELISA: enzyme-linked immunosorbent assay; epPCR: error-prone polymerase chain reaction; HA: hemeagglutinin; OST: oligosaccharyltransferase; wt: wild-type; mut: Inactive variant W458A-D459A; SDS-PAGE: sodium dodecyl sulfate–polyacrylamide gel electrophoresis; PBS: phosphate buffered saline; HRP: horseraddish peroxidase.

## Competing interests

The authors declare that they have no competing interests.

## Authors’ contributions

JI and MK designed and analyzed the experiments, jointly developed the screening methods and interpreted the results. JI drafted the manuscript. MK and MW designed and constructed the genetic systems used in the study. LW performed most mutagenesis and screening experiments and contributed significantly to methods development. RR was involved in the design of directed evolution experiments and provided crucial inputs with regard to the construction and quality control of mutant libraries. LTM participated in setting up and designing the study and contributed strongly to data interpretation and hypothesis formulation. MK, MW and LTM helped to draft the manuscript. All authors read and approved the final manuscript.

## Supplementary Material

Additional file 1 **Validation of the 96-well DWP-ELISA screening system.** Sandwich ELISA read-outs for EPA-CP5 glycoproteins and final optical densities (600 nm) of 90 wells inoculated with individual clones of *E. coli* CLM24 (pGVXN345-pGVXN150-pGVXN112Kan) (wt PglB, open symbols) and 6 distributed wells inoculated with *E. coli* CLM24 (pGVXN345-pGVXN150-pGVXN113Kan) (inactive PglB, closed symbols). ELISA absorbance at 450 nm was measured against air; OD_600_ was corrected for optical density of sterile medium. Click here for file
