# Supplementary figures and images for: Structural insights from random mutagenesis of Campylobacter jejuni oligosaccharyltransferase PglB
Source: BMC Biotechnol. 2012 Sep 24;12:67. doi: 10.1186/1472-6750-12-67 (PMC3527161; doi:10.1186/1472-6750-12-67)

## Slide 1
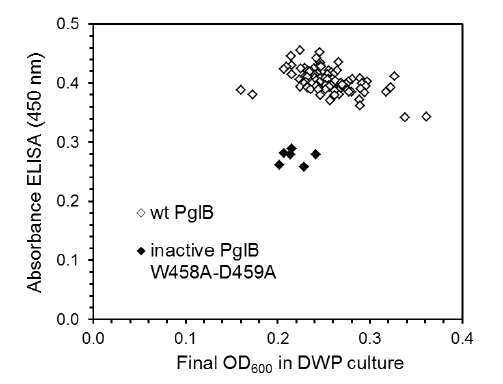

Supplement: Additional file 1 — Validation of the 96-well DWP-ELISA screening system. Sandwich ELISA read-outs for EPA-CP5 glycoproteins and final optical densities (600 nm) of 90 wells inoculated with individual clones of E. coli CLM24 (pGVXN345-pGVXN150-pGVXN112Kan) (wt PglB, open symbols) and 6 distributed wells inoculated with E. coli CLM24 (pGVXN345-pGVXN150-pGVXN113Kan) (inactive PglB, closed symbols). ELISA absorbance at 450 nm was measured against air; OD600 was corrected for optical density of sterile medium. [file 1472-6750-12-67-S1.pptx]
